# How often do surgery patients arrive in the operating room “sufficiently clean” for incision? A survey in a tertiary-care teaching hospital in France

**DOI:** 10.3205/dgkh000561

**Published:** 2025-06-26

**Authors:** Marion Lefebvre, Mélanie Consiglio Lefebvre, Sophie Notin-Coutant, Hélène Marini, Véronique Merle

**Affiliations:** 1 Department of Infection Control, Rouen University Hospital, Rouen, France; Research team “Dynamique et Evénements des Soins et des Parcours”, Le Havre, France; 2 Rouen University Hospital School for Operating Room Nurses, Rouen, France; 3 Department of Quality of Healthcare, Le Havre Hospital Group, Research team “Dynamique et Evénements des Soins et des Parcours”, Le Havre, France

**Keywords:** clinical nursing research, perioperative nursing, preoperative shower, quality of health care

## Abstract

**Aim:**

The preoperative shower (POS) is strongly recommended in skin preparation before surgery, to remove skin soiling and facilitate the antisepsis of the incision site. The effectiveness of POS to achieve clean skin in routine practice has not been studied in literature. This study aimed to determine how often the operating room (OR) nurse assessed the skin of surgical patients at arrival in the OR as “not adequately clean”.

**Method:**

This descriptive survey was carried out in January 2024 in a university hospital in France. An investigator documented patient’s age, gender, and body mass index (BMI), the day of surgery, type of surgical procedure, whether it was scheduled or unscheduled, and the site of the site. The patient was asked whether s/he had taken a POS, and its time and place (home or hospital). The OR nurse was asked by the investigator about her assessment of the patient's skin cleanliness (adequate or inadequate), and in case of skin cleanliness deemed unsatisfactory, what decision had been made: proceed as usual, additional skin cleaning in the operating room, postponement of the surgical procedure.

**Results:**

Among the 100 patients included in the study, 18% (95% CI 10.5–25.5%) had a skin cleanliness that was considered unsatisfactory. A scheduled procedure was the sole parameter significantly associated with satisfactory skin cleanliness as compared to unscheduled procedures.

**Conclusion:**

Despite the POS, approximately one-fifth of patients had inadequate skin cleanliness upon arrival in the OR, highlighting an essential area for improvement of preoperative preparation of the surgical field, especially in clean surgery.

## Zusammenfassung

**Ziel:**

Präoperatives Duschen (POD) wird in der Vorbereitung vor der Operation (OP) empfohlen. Die Wirksamkeit von POD zur Erzielung einer sauberen Haut in der Routinepraxis wurde in der Literatur nicht untersucht. In dieser Studie wurde untersucht, wie oft die Krankenschwester im Operationssaal (OP) die Haut des Patienten bei der Ankunft im OP als „unbefriedigend sauber“ einstuften.

**Methode:**

Es handelt sich um eine deskriptive Studie, die im Januar 2024 in einem Universitätsklinikum im Nordwesten Frankreichs durchgeführt wurde. Ein Untersucher erfasste Alter, Geschlecht und den Body Mass Index (BMI) des Patienten, den Tag der OP, die Art des chirurgischen Eingriffs und ob er geplant oder nicht geplant war sowie den Ort der Inzision. Der Patient wurde gefragt, ob und wenn ja, wo (zu Hause oder Krankenhaus) er poräoperative geduscht hat. Die OP-Schwester wurde vom Untersucher nach ihrem Urteil bezüglich der Hautreinheit des Patienten (zufriedenstellend oder nicht zufriedenstellend) gefragt und im Falle einer als unbefriedigend beurteilten Hautreinigung, welche Entscheidung getroffen worden war: Durchführung wie gewohnt, zusätzliche Hautreinigung im OP-Saal oder Aufschub des chirurgischen Eingriffs.

**Ergebnis:**

Von 100 in die Studie einbezogenen Patienten hatten 18% (95% confidence interval 10,5–25,5) eine als unbefriedigend empfundene Hautreinheit. Ein geplantes Verfahren war der einzige Parameter, der im Vergleich zu ungeplanten Verfahren signifikant mit einer zufriedenstellenden Hautreinheit assoziiert war.

**Schlussfolgerung:**

Trotz der Notwendigkeit des POS hatten etwa ein Fünftel der Patienten eine unzureichende Hautreinheit bei der Ankunft im OP, was einen wesentlichen Bereich für die Verbesserung der Prävention von Infektionen an der Operationsstelle hervorhebt, insbesondere bei sauberen Operationen.

## Introduction

Surgical site infections (SSI) are the third most common type of healthcare-associated infections (HAI) in France according to the 2022 French national prevalence survey, with a prevalence of 14.3% [1]. It is the leading cause of renewed operation and postoperative death, and also the main reason for litigation among HAIs. SSIs are responsible for prolonged hospital stays, cosmetic and functional sequelae, and even death [2], [3]. Given the high number of surgical procedures performed each year (5.1 million surgical stays in 2023 in France [4]), preventing SSI is a particularly important challenge. According to the World Health Organization and the Center for Disease Control and Prevention, SSI is considered to be one of the most preventable HAIs [5], [6].

Preoperative skin preparation is an important control measure, especially in clean surgery where skin represents the main source of microorganisms. In France, it is currently recommended that at least one preoperative shower be taken, using plain or antiseptic soap, the day before, or the day of the surgical procedure. The aim of this POS is to remove skin soiling to facilitate the subsequent action of the antisepsis on the incision site in the operating room. According to French 2013 guidelines [7], incision-site skin cleansing is no longer carried out systematically in the operating room (OR), but is recommended only in the presence of soiled incision-site skin. Even if the influence of preoperative washing of soiled skin on the SSI rate has not been proven by controlled studies [8] because it is not ethically feasible, soiled skin must be cleaned because antiseptics are not effective in the presence of soiling. Undoubtedly, the quality of the POS influences the quality of surgical site antisepsis carried out in the OR and is recommended in all guidelines, including those of the NICE, WHO, and CDC [5], [6], [8], [9]. 

In current practice, the patient’s skin cleanliness after the POS should normally be assessed by the surgery-ward nurse before the patient leaves for the OR, and also once more by the OR nurse upon arrival in the OR. This assessment should be performed whether the patient has showered in the hospital or at home. In the OR, if the patient's cleanliness is deemed insufficient, this assessment may lead to the decision to clean the skin while the patient is on the operating table, before antisepsis of the incision site. This unanticipated and unexpected additional cleansing would therefore disrupt the organization of the OR, requiring additional equipment. If the skin is very dirty, it may even be necessary to cancel the operation at the last minute, as the patient's position on the operating table does not lend itself well to “in-depth” cleaning. This “no go” situation due to inadequate skin preparation is identified in the “Patient safety in the operating room” checklist [10].

Boulet et al. [11] recently performed a multicenter survey consisting of interviews of patients who just had surgery about their preoperative shower. One unexpected result of that survey was that, according to patient’s report, skin cleanliness after the preoperative shower had usually not been visually assessed by caregivers, who relied on the patient’s statement that the shower had been taken. However, the authors of the previous survey were unable to examine whether this reported lack of visually assessing skin cleanliness was indeed associated with unsatisfactory skin cleanliness upon arrival in the OR.

Therefore, the aim of the present study was to estimate at what frequency the skin of surgery patients was assessed as “inadequately clean“ by the OR nurse in the OR.

## Method

A descriptive survey was carried out in a tertiary care university hospital in Northwest France. This hospital had participated in our previous survey about POS in surgery patients [11].

### Study population 

Patients included were those over 18 years of age, not institutionalized, who agreed to participate in the survey (according to French regulations, a written consent was not required), and for whom the planned incision site was cutaneous and not mucosal. Both scheduled and unscheduled procedures were included. Eye surgery and outpatient surgery were excluded.

### Data collection 

Data collection was conducted during two weeks in January 2024. Patients were included upon their arrival in the OR. For each patient corresponding to inclusion criteria, an investigator explained the purpose of the study and, if the patient agreed to participate in the survey, collected the patient’s age, gender, and body mass index (BMI), the day of surgery, type of surgical procedure and whether it was scheduled or unscheduled, as well as the site of the incision.

The patient was also asked whether s/he had taken a POS, and if so, the date, time and place (home or hospital) of the POS. The OR nurse was not present during this interview.

Once the patient was taken care of by the OR nurse, the OR nurse was asked by the investigator about her judgement regarding the patient's visible skin cleanliness (satisfactory or unsatisfactory), and in case of skin cleanliness deemed unsatisfactory, what decision had been made: to proceed as usual, perform additional skin cleaning in the operating room, or postponement of the surgical procedure.

### Analysis 

The proportion of patients arriving in the OR whose skin cleanliness was judged unsatisfactory by the OR nurse was calculated and compared according to patient’s gender, age, and BMI, to the characteristics of the surgical procedure (surgery specialty, scheduled or unscheduled procedure), and to the characteristics of the POS (performed at home or in the hospital, no shower). For comparisons, age was divided into a binary variable <75/≥75, and BMI was divided into <30/≥30 to assess if old age and obesity were associated with unsatisfactory skin cleanliness. The time between the POS and the beginning of the surgical procedure was divided into two parts based on this time quartile distribution (<third quartile/≥third quartile). Surgical specialties were grouped into “usually clean surgery” (bone and joint surgery, plastic surgery, cardio-thoracic surgery, vascular surgery) or “usually unclean surgery” (digestive surgery, urologic surgery, gynecology, ear-nose-throat surgery) according to the Altemeier classification [12] of the majority of procedures of each given surgical specialty. Incision sites were grouped in “upper body incision site” or “lower body incision site”.

Proportions were calculated together with their 95% confidence interval (95% CI). Univariate comparisons were performed using the chi-squared test or Fisher’s test, when appropriate.

The expected number of patients to be included was arbitrarily fixed at 100 patients. 

### Ethics 

All eligible patients received information on the design and the objective of the survey (French regulations do not require written consent for descriptive surveys). The local Institutional Review Board approved the study protocol (reference number is CERDE 2023/0284/OB).

## Results

One hundred patients were included in the study, as expected. Their characteristics are described in the Table 1 (Tab. 1).

Among these patients, 18 (18%; 95% CI10.5-25.5%) had unsatisfactory skin cleanliness. As shown in Table 2 (Tab. 2), the only variable significantly associated with a better skin cleanliness was a scheduled procedure vs. an unscheduled surgical procedure

There was no significant difference between patients with satisfactory and unsatisfactory skin cleanliness as regards age, gender, BMI, the place of the shower (at home or in the hospital), whether cleansing was a shower or a bedside washing, and the delay between the shower and the surgical procedure. 

It should also be noted that all patients eventually had skin cleansing in the OR, regardless of whether skin cleanliness was satisfactory or not.

## Discussion

The study showed that 18% of patients arriving at the OR had a skin cleanliness deemed unsatisfactory by the OR nurse, despite the fact that a POS had been carried out in all patients, either the day before or the day of the procedure. This would suggest, first, that these POS were not sufficiently effective, and, second, that the efficacy of POS (i.e., skin cleanliness) had not been not correctly checked by surgery ward staff before the patient left for the OR. The latter result corroborates that of our previous multicenter survey performed in surgery patients, where 79% of patients stated that their skin cleanliness after the POS had been assessed by caregivers merely by asking if the shower had been performed [11]. The present survey suggests that this method of assessing cleanliness before departure for the OR is associated with an unsatisfactory state of cleanliness upon arrival at the OR, although we cannot affirm the existence of a causal link between this non visual cleanliness assessment and unsatisfactory cleanliness in the OR due to the design of our surveys. 

To our knowledge, the quality of POS or bathing in current practice has never been evaluated, despite the fact that showering is recommended by all international guidelines [5], [8], [13]. There may be multiple reasons for unsatisfactory skin cleanliness after the POS: inadequate information of the patient regarding the importance and technique of showering, ineffective showering due to patient’s disability or to a surgery site more difficult to reach (back, foot,…), or to encrusted skin soiling in patients not used to showering. These reasons could not be assessed in the present survey, because we choose not to collect data in the ward before the shower in order to avoid changes in shower practices. 

In this survey, the only factor associated with unsatisfactory skin cleanliness after the preoperative shower was a non-scheduled surgical procedure. This is not surprising, as skin preparation is better organized when the procedure is scheduled. Indeed, in the literature, emergency surgical procedures have been shown to be associated with impaired adherence to control measures such as surgical antibiotic prophylaxis [14]. However, as unscheduled surgical procedures are also identified in the literature as associated with an increased risk of SSI, it should be an aim to improve the efficacy of POS, even for emergency procedures.

It is more surprising to see that in the present survey, operations in the “clean” category of surgical specialties were not associated with better skin cleanliness. Indeed, in “clean” surgery, such as orthopedics, SSI are usually related to the contamination of the surgery site by skin microorganisms [15], [16]. Therefore, surgical teams pay particular attention to decreasing skin bacterial load before surgery, including by bathing or showering [17]. One explanation of the unsatisfactory cleanliness of “clean” surgery patients could be that this enhanced attention of surgeons and OR nurses is not shared by the surgery ward staff in charge of supervising the preoperative shower. If confirmed, this would warrant additional information and training of surgery ward staff regarding the importance of preoperative showering in clean surgery.

In the present survey, patient characteristics were not associated with skin cleanliness after POS. Better skin cleanliness in women might have been expected, as literature suggests a slightly stricter adherence to hygiene norms in women [18], however, this was not the case here. It may be also common sense to expect impaired personal hygiene in elderly patients, because of disability. However, this has not been studied in the literature and was not retrieved in our survey. The same applies to personal hygiene in obese patients.

### Limitations

Our survey has some limitations. It was a single-center study and therefore our findings may not apply to all French health facilities. However, we included a large range of surgical specialties and various populations of patients. Due to our sample size, our survey may have not had enough power to identify some characteristics associated with unsatisfactory skin cleanliness. In addition, as explained above, we did not assess the Altemeier class of each surgical procedure, but rather classified the surgical specialties as “usually clean”, or “usually not clean”. 

The strength of our survey is that we performed an assessment of cleanliness in real conditions, i.e., upon arrival in the OR, and by the OR nurse in charge of performing this assessment in order to adequately prepare the surgery site before incision.

## Conclusions

To the authors’ knowledge, this survey is the first to assess the efficacy of the preoperative shower in real life. It allowed us to show that this efficacy is not satisfactory in nearly one-fifth of operated patients, therefore identifying a topic of interest for improvement in SSI prevention, especially in clean surgery.

## Notes

### Competing interests

The authors declare that they have no competing interests.

### Ethical approval 

All eligible patients received information on the design and the objective of the survey (French regulations do not require a written consent in such descriptive survey). The local Institutional Review Board approved the study protocol (reference number is CERDE 2023/0284/OB).

### Funding

None. 

### Acknowledgments

The authors warmly thank the students (Cléo Fernandez, Caroline Lepeltier, Clarisse Vallin and Laurence Zobel) and teachers of the Rouen Hospital School for Operating Room Nurses for their valuable help in data collection, and medical and nursing managers of the participating surgical departments and operating theatre of Rouen University Hospital for granting us access to their patients. 

### Authors’ ORCIDs

- Lefebvre M: https://orcid.org/0000-0003-3283-0431
- Merle V: https://orcid.org/0000-0002-8856-7532

## Figures and Tables

**Table 1:**
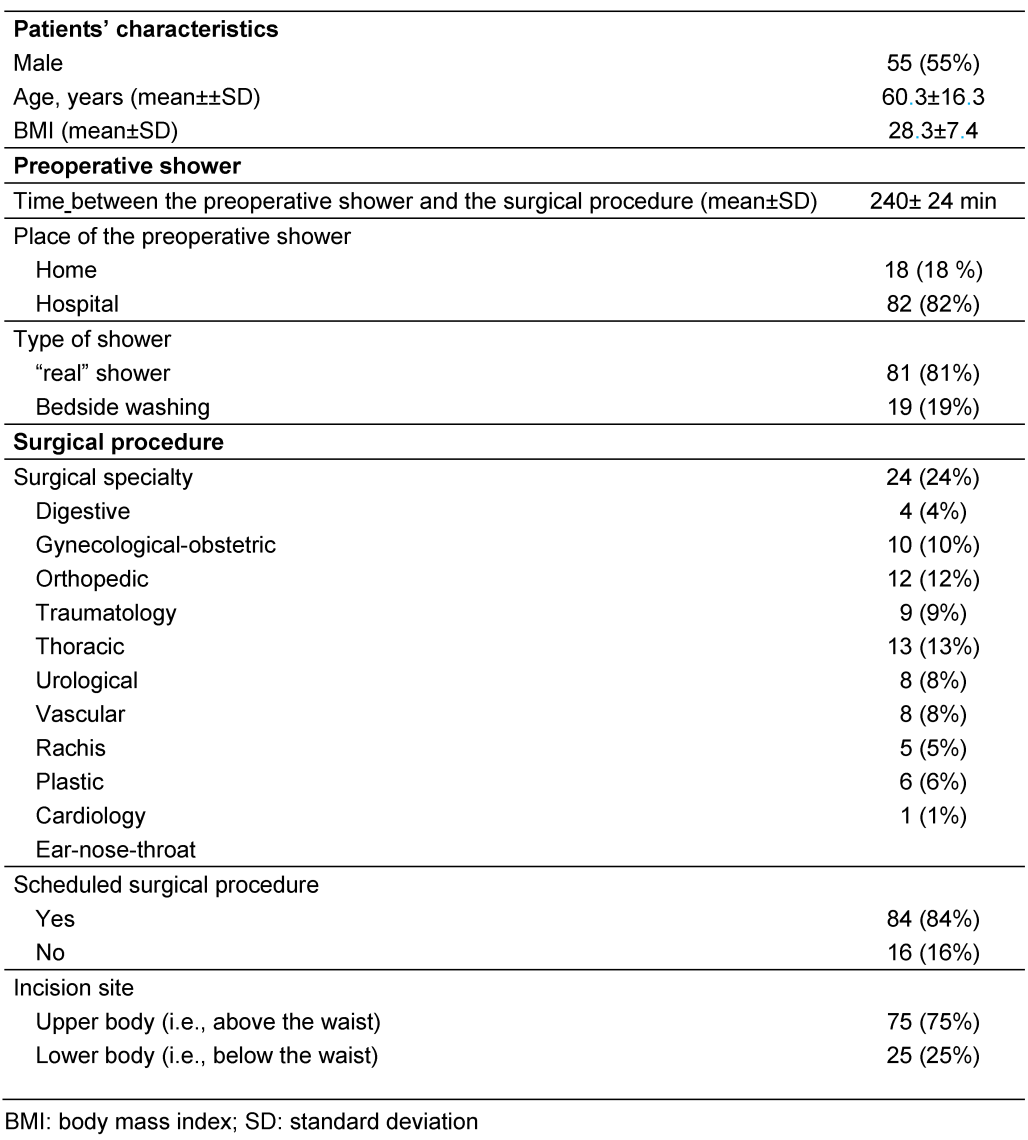
Characteristics of the survey population (n=100)

**Table 2:**
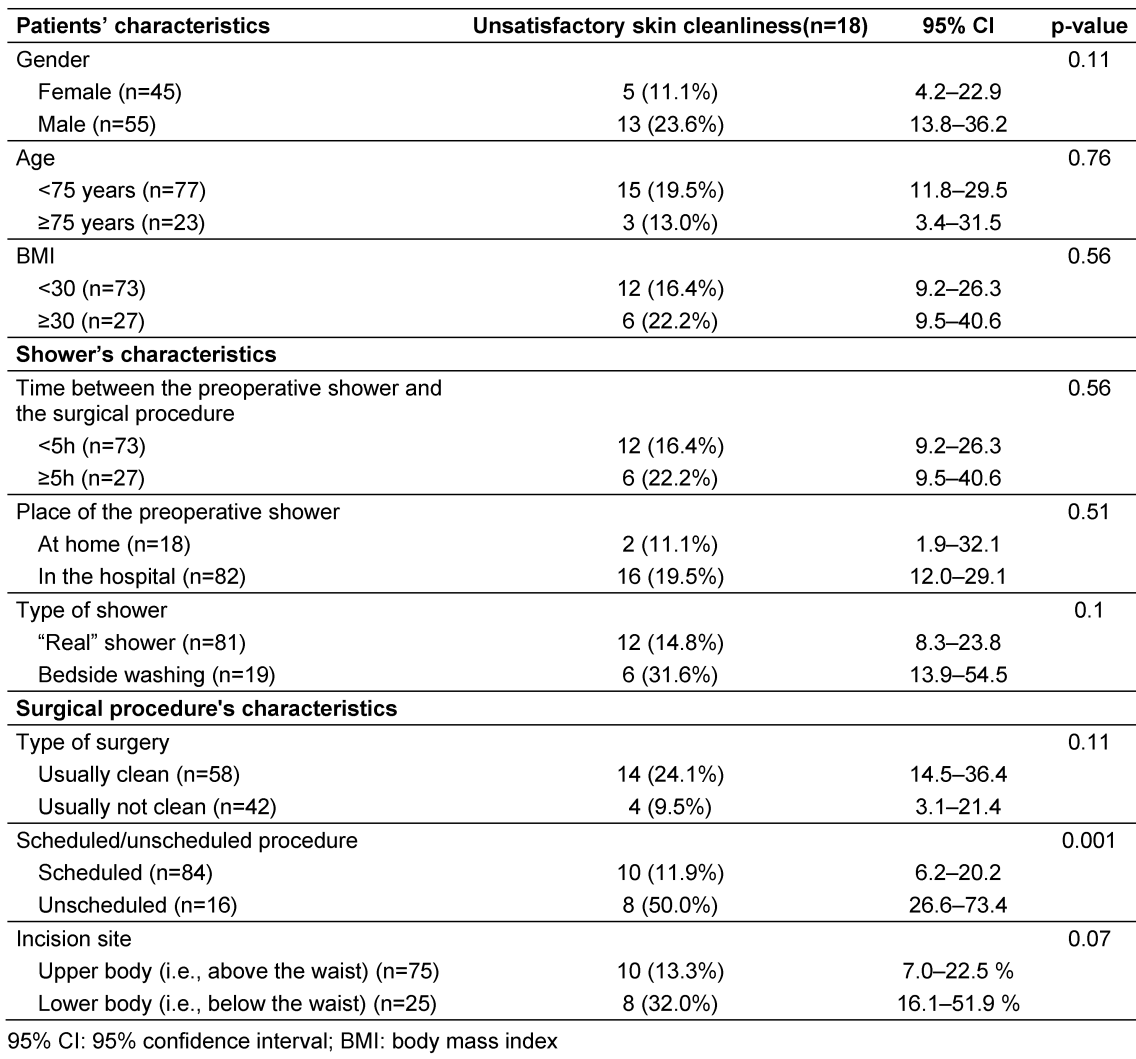
Comparison of the frequency of skin cleanliness judged unsatisfactory by the operating room nurse according to characteristics of the patient, shower, and surgical procedure
